# Antimicrobial Peptides Design Using Deep Learning and Rational Modifications: Activity in Bacteria, *Candida albicans*, and Cancer Cells

**DOI:** 10.1007/s00284-025-04346-3

**Published:** 2025-07-11

**Authors:** Andrea Mesa, Andrés Orrego, John W. Branch-Bedoya, Carlos Mera-Banguero, Sergio Orduz

**Affiliations:** 1 Departamento de Biociencias, Facultad de Ciencias, Grupo Biología Funcional, Universidad Nacional de Colombia, Sede Medellín, Carrera 65 #59A-110, Medellín, 050034 Colombia; 2 Departamento de Ciencias de la Computación y de la Decisión, Facultad de Minas, Universidad Nacional de Colombia, Sede Medellín, Avenida 80 #65-223, Medellín, 050036 Colombia; 3 Departamento de Ingeniería de Sistemas, Facultad de Ingeniería, Universidad de Antioquia, Calle 67 #53-108, Medellín, 050010 Antioquia Colombia

## Abstract

Resistance to antimicrobial agents has become a global threat, estimated to cause 10-million deaths annually by 2050. Antimicrobial peptides are emerging as an alternative and offer advantages over traditional antibiotics. Antimicrobial peptides generated by artificial intelligence (AI) strategies are potential alternatives that reduce costs and development time. This work optimized a set of peptides generated by two deep learning algorithms. The modifications made to the peptides were evaluated with bioinformatic and other AI tools as predictors of antimicrobial activity, hemolytic capacity, and toxicity. As a result, 26 synthetic peptides generated in silico were obtained with a high probability of being antimicrobial and biologically safe. Finally, 12 peptides were synthesized to perform in vitro tests against four bacterial species, *Candida albicans,* and cancer cells. Results indicate that 9 of the peptides have a MIC below 10 μM, and some have an inhibitory concentration at 2 μM, such as OrP1M for *Escherichia coli*, OrP9M for *Pseudomonas aeruginosa,* and VeP1 for *Staphylococcus aureus*. In addition, six peptides have activity against the breast cancer cell line (MCF-7), and peptide OrP1M had an IC_50_ of < 6.25 μM. It is concluded that the synthetic-generated peptides have high antimicrobial activity, but in most cases, their MICs were improved after the modifications were made.

**Supplementary Information:**

The online version contains supplementary material available at 10.1007/s00284-025-04346-3.

## Introduction

Since the discovery of antibiotics, mortality has been reduced, and significant medical advances have been achieved. However, one of the main threats to global health in the twenty-first century is the resistance of microorganisms to drugs caused by several reasons, included their inappropriate use [1]. It is estimated that by 2050, resistance will cause around 10-million deaths per year and will force nearly 24-million people into extreme poverty by 2030 [2]. Furthermore, the United States Centers for Disease Control and Prevention, estimated in 2019 that 2.8-million infections were caused by antibiotic-resistant bacteria annually, potentially causing 35.000 deaths [3].

The World Health Organization designated the species *Acinetobacter baumannii, Enterococcus faecium, Staphylococcus aureus, Klebsiella pneumoniae, Pseudomonas aeruginosa, Enterobacter* spp., *Neisseria gonorrhoeae*, and *Mycobacterium tuberculosis*, as priority species due to their high level of antibiotic resistance. It has also been found that in diseases such as tuberculosis, malaria, HIV/AIDS, pneumonia, candidiasis, and hospital infections, current pharmacologic therapies with antibiotics are not entirely successful, thus generating high transmission and mortality rates [4]. Besides, cases of antibiotic resistance have been reported in agricultural and livestock settings, leading not only to health problems but also to significant economic losses [5]. To worsen the problem, antibiotics in development are generally analogs of existing ones; of the 32 antibiotics in development, only 6 were classified as innovative [6], and the probability of success in clinical trials of new drugs for infectious diseases is 25.2%, which means a high risk of failure [4, 7]. The high costs and time invested for antibiotic development in the pharmaceutical industry produce lower revenues, which has prompted companies to develop drugs with a higher return on investment [7].

As an alternative to antibiotics, antimicrobial peptides (AMPs) have been studied as new alternatives against drug-resistant pathogens [5]. AMPs are evolutionarily conserved molecules of between 5 to 50 amino acids that play critical roles in the innate immunity and can be found naturally in bacteria, protists, plants, fungi, invertebrates, and vertebrates [1, 5]. Unlike conventional antibiotics, AMPs alter the cell membrane by binding to anionic surfaces, facilitating hydrophobic interactions and causing cell death through various mechanisms. This mode of action hinders the development of resistance due to the high cost of changing cell membrane components [8].

Furthermore, specific characteristics of AMPs, such as amino acid composition, charge, amphipathicity, and hydrophobic moment, among others, favor the interaction and pore formation in the lipid bilayer of the plasma membrane, leading to cell death [9]. Despite the therapeutic potential of AMPs, there are limitations, such as the high cost and time required to discover new AMPs in classical bioprospecting processes. Among the main limitations for their development are the length of the AMP sequences, which entails high production costs on an industrial scale, toxicity, susceptibility to proteolytic degradation, low stability, and poor pharmacokinetics. The design of AMPs must consider improvements in methodologies and strategies to optimize their efficacy, specificity, and therapeutic potential, both in natural and synthetic peptides, to compete more effectively in the market. New bioprospecting strategies include rational design, combinatorial chemistry, structural modification, and a greater understanding of the physicochemical-activity relationship [10].

Although around 5000 AMPs have been reported in nature, some have low antimicrobial potential and lengths greater than 30 amino acids, which hinders their potential use due to their high synthesis cost and strenuous work for isolation and experimental testing [11]. To reduce the costs and development time, artificial intelligence based on deep learning algorithms has been used to generate de novo synthetic AMPs [12, 13], improving their activity by bioengineering, which is a promising new low-cost strategy to obtain short peptide sequences with ideal pharmacologic characteristics in a few months [14, 15]. Following this line of research, this study used artificial intelligence strategies based on deep learning to generate synthetic peptide sequences, which were optimized and then experimentally validated against *Candida albicans* and four clinically critical bacterial species with reported resistance. Their cytotoxic activity was also evaluated against MCF-7 and A549 cancer cell lines.

## Materials and methods

### Generation and modification of synthetic antimicrobial peptides

Peptide sequences were generated with PepGen 1.0 [13] and AmPepGen [16]. PepGen 1.0 is a synthetic peptide generator trained with a recurrent neural network (RNN) with LSTM (*Long Short-Term Memory*) cells, and was used to generate 3006 peptide sequences with lengths between 12 and 20 amino acids. On the other hand, AmPepGen [16] is an antimicrobial peptide generator trained with GAN (Generative Adversarial Network), a deep learning architecture. AmPepGen was used to generate 2998 peptide sequences with lengths between 7 and 35 amino acids, for a total of 6004 peptides generated. PepGen 1.0 can be freely accessed at https://bit.ly/2Z281cY, and the repository of the AmPepGen generator [16] is available at https: //github.com/Anorpe/ampepgen-dev.

**Fig. 1:**
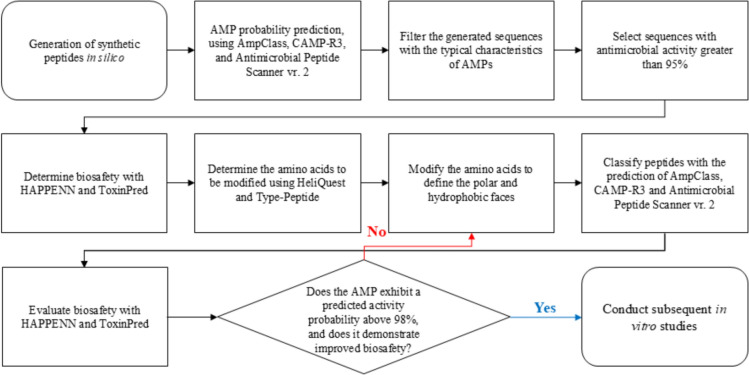
Flowchart of the strategy used in the *in-silico* design of synthetic peptides generated by AI with antimicrobial potential

The antimicrobial peptide sequences were selected and optimized according to the flow chart illustrated in Fig. 1. Those peptides with a high probability of having antimicrobial activity greater than or equal to 95% were selected, according to the peptide classification models available in CAMPR3 [17], AMP Scanner Vr.2 [18] and AmpClass 1.0 [19]. The top six peptides were modified to increase their probability of antimicrobial activity so that it was greater than or equal to 98%. The hydrophobic and hydrophilic faces were improved using Heliquest [20] and Type-Peptide [21] (Figure S2). Peptide biosafety predictions related to toxicity and hemolytic activity were determined with ToxinPred [22] and HAPPENN [23], respectively. Subsequently, the similarity of the selected and modified peptide sequences was determined with the AMPs available in APD3, Antimicrobial Peptide Database [11].

The anticancer activity was predicted with ENNAACT [24], while the membrane binding probability of the peptides was estimated using AmphipaSeeK, which provides a score between -1 and 1 for each amino acid, where a value greater than 0.5 represents a high binding probability [25]. The results obtained are presented in Table S1 of the supplementary information.

A random peptide was selected as the negative control, while the synthetic peptide SlP20, reported by Monsalve et al. [15] and experimentally validated for its activity against various clinically relevant pathogens, was used as the positive control. Both controls were subjected to activity predictions using all previously employed algorithms.

The 12 resulting peptides (the six selected and their modified versions) were synthesized by Biomatik (Wilmington, Delaware, USA) with a minimum purity of 70% and characterized by mass spectrometry (Table S2).

### Experimental validation of antimicrobial activity

The antimicrobial activity of the peptides was evaluated with *S. aureus* ATCC 25923, *P. aeruginosa* ATCC 27853, *Escherichia coli* ATCC 25922, *Klebsiella quasipneumoniae* ATCC 700603, and the yeast *C. albicans* ATCC 10231. The minimum inhibitory concentration (MIC) was determined following the protocol described by Wiegand et al. [26]. Briefly, bacteria were grown overnight in Mueller–Hinton (MH) medium at 37 °C and 90 rpm, while *C. albicans* was grown at 30 °C in MH supplemented with 30% YPD (yeast extract, peptone, dextrose). The concentration of bacteria and *C. albicans* was adjusted to 10^6^ CFU/mL. Each peptide was diluted in 96-well plates to obtain final concentrations of 128, 64, 32, 16, 8, 8, 4, 2, and 1 µM. Microorganism growth was measured hourly at 600 nm for 24 h, with a 10-s shaking cycle, on a Multiskan Go microplate reader (Thermo Scientific). The experiments with bacteria were incubated at 37 °C, whereas those with *C. albicans* were incubated at 30 °C. Each peptide was evaluated by duplicate on 2 different days. The MIC was estimated as the minimum peptide concentration at which the microorganism had no growth.

As a positive control for bacterial inhibition, Penicillin–Streptomycin (Pen-Strep; 10,000 units/mL penicillin and 10,000 µg/mL streptomycin) was used at a final concentration of 50%. For *C. albicans*, 5% Triton X-100 was employed as the positive control. In all assays, untreated culture medium without peptides was included as a negative control to account for basal microbial growth. All assays were performed in two independent experiments, each conducted with two technical replicates (*n* = 4 per condition).

### Cytotoxic activity against cancer cells

The methodology proposed by Zhang et al. [27] was followed with human lung adenocarcinoma (A549) and human breast adenocarcinoma (MCF-7) cells, which were cultured in Roswell Park Memorial Institute (RPMI 1640) medium supplemented with 10% fetal bovine serum (FBS) and 1% streptomycin and penicillin. The cells were maintained at 37 °C, 1 atmosphere with 5% CO_2_ until a confluence greater than 80% was achieved. The inhibition of cancer cell growth and the cytotoxicity of the peptides were evaluated using the MTT assay (3-(3,4-dimethylthiazol-2-yl)-2,5-diphenyltetrazolium bromide), for which cell densities of 1.0 × 10^4^ for MCF-7 and 1.5 × 10^4^ for A549 were seeded in each well of a 96-well plate and incubated for 24 h to allow proper cell adhesion. Then, 10 μL of each peptide were added to obtain final concentrations of 50, 25, 12.5, and 6.25 μM by triplicate. After 24 h of incubation, the wells were washed twice with PBS, and 10 μL of MTT solution was added to each well at a final concentration of 0.5 mg/mL, and the cell cultures were incubated for 4 h at 37 °C. The solution was removed from each well, and the formazan crystals were dissolved with 100 μL of Sodium Dodecyl Sulfate (0.01 M). After 16 h, absorbance was measured with a MultiSkan Go microplate reader at 570 nm. The untreated wells were used as a negative control, while wells treated with DMSO were used as a positive control for cytotoxicity. The percent viability was determined using the following equation.

$$\% {\text{viability}} = \frac{{{\text{ABS}}_{M} - {\text{ABS}}_{C - } }}{{{\text{ABS}}_{C + } - {\text{ABS}}_{C - } }} \times 100\%$$

where ABS_M_ is the average absorbance with the treatment and ABS_C_ is the absorbance obtained in the controls (positive and negative). With these data a non-linear regression was generated, and the half inhibitory concentration (IC_50_) determined using Excel (version 17.0). The half inhibitory concentration is the peptide concentration required to inhibit cell viability by 50%.

### Hemolysis assays

Human blood from a healthy fasting volunteer donor was collected in sodium citrate tubes and centrifuged at 1000 XG at room temperature for 7 min. The sediment was washed 3 times with sterile 0.9% phosphate buffered saline (PBS), centrifuged at 1000 XG and the supernatants were discarded. A 1:10 suspension of erythrocytes in 0.9% PBS solution was prepared while mixing 90 µL of the diluted erythrocyte suspension with 10 µL of each peptide in Eppendorf tubes to obtain peptide concentrations of 50, 25, 12.5, 6.25, and 3.13 µM. The treated erythrocytes were incubated at 37 °C for 3 h at 90 rpm and centrifuged at 1000 XG at room temperature for 5 min. Fifty µL of the supernatants were transferred to a 96-well plate, and the absorbances were measured at 545 nm. Triton X-100 (0.1%) was used as a positive control, and sterile 0.9% PBS as a negative control [27]. Each treatment had three replicate wells, and the percentage of hemolysis was calculated at 50 µM for each peptide using the following equation.

$$\% {\text{Hemolysis}} = \frac{{{\text{OD}}\left( {{\text{PEP}}} \right) - {\text{OD}}\left( {0.9\% {\text{PBS}}} \right)}}{{{\text{OD}}\left( {0.1\% {\text{Triton}} \times 100} \right) - {\text{OD}}\left( {0.9\% {\text{PBS}}} \right)}} \times 100\%$$

where OD(x) is the optical density of each solution at 545 nm. The minimum hemolytic concentration (MHC) was determined as the lowest peptide concentration that induced 10% hemolysis of human red blood cells.

### Therapeutic index

The Therapeutic Index (TI) was calculated for each microbial species and peptide as TI = peptide concentration required to induce 10% hemolysis/MIC. High TI values indicate that the peptide causes low hemolysis and is highly active against microbial species at low concentrations [28]. The hemolytic activity curve was used to interpolate the minimum peptide concentration that results in 10% hemolysis (MHC) [29].

### Cell morphology using scanning electron microscopy

The effect of the peptides on cell morphology was observed using scanning electron microscopy with two peptides with the best MICs against each microorganism, according to the protocol of Marcellini et al. [30]. Bacterial cultures were grown in MH broth at 37 °C and 90 rpm, while *C. albicans* was grown at 30 °C in MH supplemented with 30% YPD. The concentration of all microbial species was adjusted to 10^6^ CFU/mL, corresponding to an optical density of 0.09–0.1 at 600 nm. Subsequently, 100 µL of the culture were placed in Eppendorf tubes and mixed with 100 µL of the peptide to achieve a final concentration of twice its MIC for each microbial strain and peptide. The treatments with bacterial cultures were incubated at 37 °C and 90 rpm for 2 h, while treatments with *C. albicans* were incubated at 30 °C for 4 h. Subsequently, each treatment was vacuum filtered with 0.21-µm cellulose acetate membranes (Sartorius AG, Germany), and the microbes were fixed with 2.5% v/v glutaraldehyde solution for 2 h. The cells were dehydrated in increasing ethanol concentrations of 30, 50, 70, and 96% v/v, each for 5 min. The filters were air-dried for 24 h, and samples were then coated with a thin layer of gold in a Denton Vacuum Desk IV (Denton Vacuum, LLC-USA) and observed using a JSM 6490 LV scanning electron microscope (JEOL, USA) under vacuum. The secondary electron detector was used to observe the topography and morphology of the bacterial cells.

### Statistical analysis of antimicrobial activity

To assess whether the structural modifications introduced into the peptides led to statistically significant differences in antimicrobial activity, a one-way analysis of variance (ANOVA) was conducted. This analysis was performed on the MIC values obtained experimentally for each peptide variant. Each original and modified peptide was tested in four independent replicates against each of five target pathogenic microorganisms (*C. albicans*, *E. coli, K. quasipneumoniae, S. aureus*, and *P. aeruginosa*).The data were grouped according to peptide type (original vs. modified) and analyzed to compare MIC distributions using ANOVA. A *P* value < 0.05 was considered indicative of statistical significance. All statistical analyses were performed using R version 4.3.2 (R Core Team, 2023).

A summary of the statistical comparisons is provided in Table S5, highlighting the significance levels for each peptide–microorganism combination and indicating where peptide modification resulted in improved activity.

## Results

### Generation and modification of synthetic antimicrobial peptides

Six thousand four (6004) synthetic peptides with antimicrobial potential were generated using PepGen 1.0 [13] and AmPepGen [16]. The antimicrobial probability of those peptides was estimated using AmpClass 1.0, CAMPR3, and Antimicrobial Peptide Scanner Vr.2 tools. Thirteen of those peptides showed a probability of being antimicrobial equal to or greater than 95% and the 6 peptides with the highest probabilities were selected and modified to enhance their antimicrobial potential, hydrophobic and hydrophilic faces, and biosafety profile (Table 1).

**Table 1:** Description of physicochemical characteristics of synthetic peptides generated with PepGen 1.0, AmPepGen and their modified versions, with predictions of their antimicrobial activity and biosafety using machine learning algorithms

Peptide name^*^	Peptide sequence	% H^a^	Net charge^a^	Boman Index^a^ (kcal/ mol)	Prediction of antimicrobial activity^b^				Biosafety prediction	
					AMP Scanner	CAMP-R3	AmpClass		Toxin Pred	HAPPENN
					DNN	RFC	RF	XG	SVM	ANN
OrP1	AGLARKWLKLPIGVLAR	54	4	0.16	0.99	0.99	0.96	1.00	− 1.35	0.01
OrP1M	LAKRWLKLLGKLAK	57	5	0.40	1.00	0.99	0.99	1.00	− 0.21	0.12
OrP3	AGLKKLAKKLGKVVL	46	5	− 0.37	0.99	0.96	0.99	1.00	− 1.11	0.04
OrP3M	ALRKLLKKLAKAVKA	60	6	0.78	1.00	0.97	1.00	1.00	− 1.19	0.04
OrP4	AKLLGKLLRKKIGVL	57	5	− 0.01	0.99	0.97	1.00	1.00	− 1.36	0.03
OrP4M	LKRLAKLLKKWIKA	57	6	0.87	1.00	0.99	1.00	1.00	− 0.25	0.04
OrP9	FKLFCVAKRLPKKVL	53	5	0.35	0.99	0.98	0.98	1.00	− 0.40	0.14
OrP9M	IGKVWKVAKKLIKI	57	5	− 0.36	1.00	0.99	1.00	1.00	− 1.14	0.02
VeP1	FKKLLKFLKWLF	59	4	− 0.73	0.99	0.95	1.00	1.00	− 0.79	0.87
VeP1M	IKKWGKVLKKLI	50	5	0.06	1.00	0.98	1.00	1.00	− 1.35	0.03
VeP2	IKPVVKGIKGVKNFFK	55	5	0.29	0.99	0.99	0.97	1.00	− 1.18	0.03
VeP2M	AIVKKIGKIWKNFIK	53	5	0.14	1.00	0.99	0.99	1.00	− 1.15	0.09
Random^+^	SELVTTPCPVPHSH	29	− 1	0.98	0.27	0.13	0.50	N.A	− 1.00	N.A
SlP20*	GFLARKALRALKGLVKL	59	5	0.44	0.99	0.99	0.99	1.00	− 1.21	0.06

^*^The letter M in the name of the peptides indicates that the sequence was modified by rational design

^a^Parameters calculated in APD3: Antimicrobial Peptides Database[11]

^**b**^Prediction of antimicrobial activity estimated with algorithms available in CAMPR3 [17], AMP Scanner Vr2 [18] and AmpClass[19]

^**c**^Biosafety prediction estimated using algorithms available on ToxinPred [22] and HAPPENN [23] platforms respectively

Abbreviations: %H, hydrophobic percentage; DNN, Deep neural network; RFC, random forest classifier; RF; random forest; XG, XGboost; SVM, support vector machine; ANN, artificial neural network; AMP, antimicrobial peptide. N.A. Not available

^+^Randomly generated peptide, employed as a negative control

^*^Peptide derived from Monsalve et al. [15], exhibiting experimentally validated biological activity, employed as a positive control

The complete information of the six selected peptides and the specific modifications made to their sequences are shown in Table S3. Four peptides were selected from the AmPepGen generator (OrP) and two from PepGen 1.0 (VeP). Modifications of the peptide sequences increased the likelihood of being antimicrobial and decreased the prediction of hemolysis and toxicity to mammalian cells. Moreover, the evaluated peptides exhibited activity predictions comparable to those of the positive control, in sharp contrast to the negative control peptide (random), which showed considerably low predicted activity.

On the other hand, the alignment of the 12 sequences with the peptides reported in APD3 [11] indicates that they are similar to other natural and synthetic peptides with experimentally validated antimicrobial, antibacterial, antifungal, and anticancer activity in the 47.4 to 66.7% range. Refer to Table S3 and Figure S3 in the supplementary information to see the details.

### Antimicrobial activity

As shown in Table 2, all selected and modified peptides demonstrated activity against at least 1 bacterial species or *C. albicans* at 16 μM or lower concentrations, except for peptide VeP2. Seventy five% of the peptides tested (i.e., 9) showed MICs under 10 μM against at least 1 bacterial species. Of these, 7 peptides demonstrated activity against at least 2 bacteria, and OrP4M showed activity against all 4 bacteria, with MICs ranging from 3.5 to 7.1 µM. In addition, three generated peptides (OrP3, OrP9, and VeP1) exhibited a MIC lower than 16 µM against at least 2 bacterial species. On the other hand, the six modified peptides showed a MIC equal to or less than 16 µM against at least 4 microorganisms, with three showing activity against all 5 microorganisms below 16 µM. Seven peptides were active against *C. albicans* with MICs below 16 µM. Notably, peptide OrP9M showed activity against two bacterial species and *C. albicans* with MICs below 10 µM. On the other hand, peptide VeP1 exhibited the lowest MIC, 1.8 µM, against *S. aureus*, while OrP1M and OrP9M had MICs of 1.9 µM against *E. coli* and *P. aeruginosa*, respectively.

**Table 2:** Biologic activity of computer generated and modified peptides against five bacterial species, *Candida albicans*, red blood cells, and cancer cell lines

Peptide name^a^	Sequence	MIC* (µM)					% Hemolysis at 50 µM	IC_50_ (µM)	
		C.a	E.c	K.q	S.a	P.a		A549	MCF-7
		MIC (MFC)	MIC (MBC)	MIC (MBC)	MIC (MBC)	MIC (MBC)			
OrP1	AGLARKWLKLPIGVLAR	24.8 (49.7)	12.4 (12.4)	49.7 (> 99.3)	> 99.3 (> 99.3)	49.7 (99.3)	1.2	21.7	9.1
OrP1M	LAKRWLKLLGKLAK	15.8 (31.6)	1.9 (1.9)	15.8 (15.8)	3.9 (3.9)	3.9 (3.9)	6.8	21.1	< 6.25
OrP3	AGLKKLAKKLGKVVL	13.9 (27.8)	3.5 (3.5)	13.9 (27.8)	27.8 (27.8)	13.9 (13.9)	0.0	33.1	23.2
OrP3M	ALRKLLKKLAKAVKA	13.3 (13.3)	3.3 (6.6)	26.6 (> 106.4)	13.3 (13.3)	3.3 (3.3)	1.0	18.1	7.4
OrP4	AKLLGKLLRKKIGVL	62.6 (125.3)	31.3 (62.6)	> 125.2 (> 125.2)	> 125.2 (> 125.2)	15.7 (31.3)	20.9	> 100	> 100
OrP4M	LKRLAKLLKKWIKA	14.1 (28.2)	3.5 (3.5)	7.1 (14.1)	7.1 (14.1)	3.5 (3.5)	9.5	15.7	< 6.25
OrP9	FKLFCVAKRLPKKVL	> 119.2 (> 119.2)	7.5 (7.5)	> 119.2 (> 119.2)	> 119.2 (> 119.2)	14.9 (29.8)	0.0	> 100	> 100
OrP9M	IGKVWKVAKKLIKI	7.8 (15.6)	7.8 (7.8)	15.6 (15.6)	15.6 (31.1)	1.9 (1.9)	2.3	25.1	< 6.25
VeP1	FKKLLKFLKWLF	56.6 (> 113.1)	7.1 (14.1)	28.3 (28.3)	1.8 (3.5)	56.6 (56.6)	55.6	6.4	6.8
VeP1M	IKKWGKVLKKLI	15.2 (30.3)	7.6 (7.6)	30.4 (30.4)	15.2 (15.2)	3.8 (3.8)	0.0	35.0	13.6
VeP2	IKPVVKGIKGVKNFFK	51.5 (> 102.9)	102.9 (102.9)	102.9 (> 102.9)	51.5 (> 102.9)	51.5 (102.9)	3.0	69.6	34.2
VeP2M	AIVKKIGKIWKNFIK	15.6 (31.3)	3.9 (3.9)	31.3 (62.6)	15.6 (31.3)	7.8 (15.6)	7.1	34.9	21.9

^a^ The letter M in the name of the peptides indicate that the sequence was modified by rational design

^*^MIC, Minimum inhibitory concentration; MFC, Minimum fungicide concentration; MBC, Minimum bactericidal concentration; IC_50_, Half inhibitory concentration

Abbreviations of the microorganism species used: C.a., *Candida albicans*; E.c., *Escherichia coli*; K.q., *Klebsiella quasipneumoniae*; S.a., *Staphylococcus aureus*; P.a., *Pseudomonas aeruginosa*

Also, 9 peptides were active against *E. coli* in a concentration range of 1.9 to 7.8 µM, 6 peptides were active against *P. aeruginosa* in the same concentration range, 3 peptides showed activity against *S. aureus* with MICs in a range of 1.8 to 7.1 µM, and peptide OrP4 was active against *K. quasipneumoniae* at a concentration of 7.1 µM. Six of the peptides were found to have a minimum bactericidal concentration (MBC) equal to the MIC, with values below 16 µM against at least 2o bacterial species. For *C. albicans*, only peptides OrP3M and OrP9M showed a minimum fungicidal concentration (MFC) lower than 16 µM (Table 2).

### Cytotoxic activity against cancer cells

In the MCF-7 cell line, 6 of the 12 evaluated peptides showed activity with half inhibitory concentrations (IC_50_) below 20 μM (in the range of < 6.25 to 13.6 μM). In addition, 3 peptides showed activity against A549 cells, with IC_50_ in the 6.4 to 18.1-μM range. On the other hand, peptides OrP3M, OrP4M, and VeP1 showed activity against both cell lines, with IC_50_ in the range of 6.4 to 18.1 μM and < 6.25 to 7.4 μM against A549 and MCF-7, respectively. While peptides OrP1, OrP1M, OrP3M, OrP4M, OrP9M, VeP1, and VeP1M exhibited activity with IC_50_ in the range of < 6.25 to 13.6 μM on MCF-7. In contrast, peptides OrP4 and OrP9 did not show cytotoxicity at 100 μM, the highest concentration evaluated. It is observed that, in general, the modified peptides had a lower IC_50_ in both cell lines compared to the originals, except peptide VeP1.

### Hemolysis and therapeutic index

Table 3 contains the minimum hemolytic concentration of synthetic peptides in human red blood cells and their TI calculated for each microbial species. Accordingly, 10 peptides did not cause hemolysis in red blood cells exceeding 10% at 50 µM, along with MICs lower than 16 µM against at least 1 microbial species. Six of these peptides showed MICs below 10 µM against 2 bacterial species, with a hemolysis range from 0.0 to 9.5% at 50 µM. Only peptides VeP1 and OrP4 caused significant hemolysis (> 10%) in human erythrocytes, reaching 20.9% and 55.6% at 50 µM, respectively. When comparing the hemolysis results with the antimicrobial activity, it was observed that the highest TI was 52.6 for peptides OrP1M with *E. coli* and OrP9M with *P. aeruginosa*. In general, peptide OrP1M displays a TI in the range of 25.6 to 52.6 for three bacterial species, while OrP3M presents a TI of 30.3 for two bacterial species. The most effective peptide against *C. albicans* was OrP9M, with a TI of 12.8.

**Table 3:** Minimum hemolytic concentration of synthetic peptides in human red blood cells and Therapeutic Index calculated for each microbial species and peptide

Peptide name^a^	MHC^b^ (μM)	Therapeutic Index (TI^c^) when tested on				
		C.a	E.c	K.q	S.a	P.a
OrP1	*	4.0	8.1	2.0	–	2.0
OrP1M	*	6.3	52.6	6.3	25.6	25.6
OrP3	*	7.2	28.6	7.2	3.6	7.2
OrP3M	*	7.5	30.3	3.8	7.5	30.3
OrP4	28.98	0.5	0.9	–	–	1.8
OrP4M	59.91	4.2	17.1	8.4	8.4	17.1
OrP9	*	–	13.3	–	–	6.7
OrP9M	*	12.8	12.8	6.4	6.4	52.6
VeP1	4.25	0.1	0.6	0.2	2.4	0.1
VeP1M	*	6.6	13.2	3.3	6.6	26.3
VeP2	*	1.9	1.0	1.0	1.9	1.9
VeP2M	68.64	4.4	17.6	2.2	4.4	8.8

^a^M in the name of the peptides indicate that the sequence was modified by rational design

^b^MHC: Minimum hemolytic concentration

^c^TI = MHC / MIC, where the MIC is the minimum inhibitory concentration

^*^For some of the peptides, hemolytic activity assays did not achieve hemolysis greater than 10% at the evaluated concentrations; therefore, a value of 100 μM was used to calculate the therapeutic indices

Abbreviations of microorganism species: C.a., *Candida albicans*; E.c., *Escherichia coli*; K.q., *Klebsiella quasipneumoniae*; S.a., *Staphylococcus aureus*; P.a., *Pseudomonas aeruginosa*

### Effect of antimicrobial peptides on microbial cell morphology

**Fig. 2:**
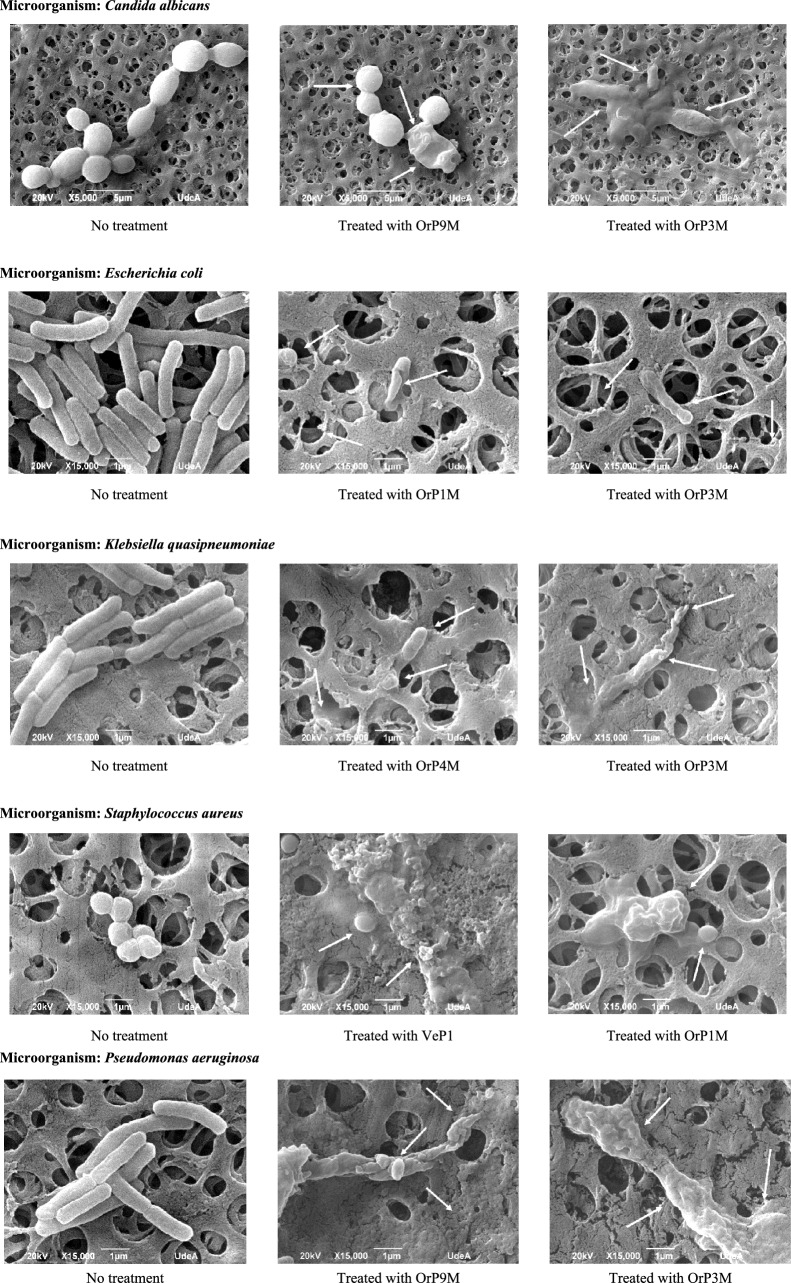
Scanning electron microscopy of *C. albicans, E. coli, K. quasipneumoniae, P. aeruginosa* and *S. aureus* cells treated with antimicrobial peptides at twice their respective MICs. Arrows indicate cell damage and release of cytoplasmic material

Figure 2 presents the scanning electron microscopy images of *C. albicans, E. coli, K. quasipneumoniae, P. aeruginosa,* and *S. aureus* cells with and without treatment with AMPs at twice their respective MICs. Images in Fig. 2 show that *C. albicans* and the untreated bacterial species have standard sizes, smooth and turgid surfaces, with rod or spherical shapes, according to the expected morphology for each species, while the microorganisms treated with the two most active peptides for each species, at a concentration 2X of their respective MICs for 2 h, showed smaller size, with alterations in the shape and structure of the cell membrane. These changes include membrane wrinkling, vesicle and groove formation, cell destruction, and release of cytoplasmic material.

*C. albicans* treated with peptide OrP9M caused loss of turgor and release of cytoplasmic material, while with OrP3M, loss of membrane structure with release of cytoplasmic material was observed. The treatment of *E. coli* with OrP1M and OrP3M shows membrane wrinkling, groove formation, and cell destruction. Peptide OrP4M in *K. quasipneumoniae* causes vesicle formation ranging from 0.1 to 0.5 μm in diameter, while OrP3M causes cell disintegration and cytoplasmic material release. In the case of *S. aureus*, treatments with peptides OrP1M and VeP1 led to a significant reduction in cell diameter, from 0.8 to 1 μM in the control to 0.5 to 0.7 μM in the treatments, with loss of characteristic cell morphology; moreover, in the case of VeP1, cell destruction was observed with leakage of cytoplasmic material. On the other hand, *P. aeruginosa* treated with OrP9M showed blisters formation ranging from 0.2 to 0.5 μm in diameter along with loss of normal cell shape. At the same time, treatment of *P. aeruginosa* with peptide OrP3M resulted in greater cytoplasmic material leakage with complete disintegration of the cell membrane. In all peptide treatments, a significant decrease in the number of cells is observed compared to the controls.

## Discussion

Antimicrobial peptides stand out as next-generation antibiotics due to their activity at low concentrations, low toxicity, broad spectrum, and therapeutic potential. However, traditional experimental approaches to discover new AMPs are time and money-demanding [31]. Recently, interest in computer-aided peptide design has increased, using linguistic models and machine learning techniques that reduce costs and time spent developing peptides with specific properties, improving their stability, biologic activity, and specificity [32]. These predictive models, experimentally validated, have allowed the generation of AMPs with an efficiency of up to 82% [12]. Likewise, recurrent neural networks have made possible to obtain highly effective peptides against cells such as MCF7, without affecting human erythrocytes [33]. However, the combinatorial approach of artificial intelligence-based techniques together with bioengineering-guided modifications has shown promise in obtaining peptides with high biologic potential at a low cost and in a short time [14, 15].

In this study, a combinatorial computational strategy was validated using artificial intelligence methods based on neural networks to generate peptide sequences with high biologic potential and performing rational modifications in the generated sequences. Three thousand six peptides were generated with PepGen 1.0, which employs RNNs with LSTM cells, and 2998 were generated with AmPepGen, a generative artificial neural network [16]. The antimicrobial probability of the generated peptides was estimated using the algorithms included in AmpClass 1.0, AMP Scanner Vr.2, and CAMPR3. On the other hand, ToxinPred and HAPPENN were used to predict their biosafety. Thirteen peptides were identified with a probability of being antimicrobial and neither toxic nor hemolytic. The six with the highest probability of having antimicrobial activity were selected and modified to increase their antimicrobial activity while maintaining the characteristic parameters of being AMPs (Tables 1 and 2).

The 12 peptides (the six original and their modifications) have a molecular weight between 1454 and 1862 Da (12 to 17 amino acids in length) and a net charge between + 4 and + 6, higher than the average value of + 2 observed in AMPs documented in the literature, and lower than + 8, which is associated with a higher probability of hemolytic activity [34]. The Boman index, which measures the average free energy of the amino acid side chains of a peptide required to transfer from cyclohexane to water [35], fell within the range of -0.73 to 0.87; a Boman index close to 0 indicates a higher probability of interaction with the lipids of bacterial membranes.

As shown in Table 1, the selected and modified peptides exhibited significantly higher activity predictions compared to the negative control, a randomly generated peptide. These results demonstrate that the generators employed in this study are capable of producing peptide sequences with a high probability of biologic activity. In fact, several of the designed peptides achieved prediction scores comparable to those obtained for experimentally validated peptides using artificial intelligence algorithms, as observed with the SlP20 peptide used as a positive control, which was developed through different methodologies.

The secondary structure of all peptides was predicted using AlphaFold2 [36], and in all cases, a pLDDT (predicted local distance difference test) greater than 70 was obtained, indicating that high-confidence models were generated for the prediction of the peptide structures. In addition, an alpha helix prediction was observed for all peptides except for OrP3, which showed an unstructured model, as shown in Figure S1 of the supplementary material.

The prediction values obtained with ToxinPred’s Support Vector Machine algorithm ranged from − 1.36 to − 0.21, indicating that all peptides have a very low probability of being toxic to mammalian cells [22]. In contrast, the HAPPENN neural network algorithm uses a normalized sigmoid score probability ranging from 0 to 1, where 0 indicates a very low probability of being hemolytic and 1 a high probability. The prediction values for the modified peptides ranged from 0.01 to 0.12, suggesting that they have a low probability of affecting red blood cells [23]. The prediction of hemolytic activity was greater than 10% for the peptides OrP1M, OrP9, and VeP1. However, the in vitro results showed that only the peptide VeP1 exhibited significant hemolytic activity at 50 µM (55.6%), while the other two peptides maintained a hemolysis percentage below 10%, considered safe. On the other hand, OrP4 presented an in-silico prediction of 3% hemolysis with HAPPENN, but upon experimental verification, the peptide caused 20.9% hemolysis at a concentration of 50 µM (Tables 1 and 3).

The sequence of the peptides was compared with the AMPs from APD3 [11], finding that they share similarity percentages between 47.4% and 66.7%, mainly with synthetic peptides such as DP1, Hp-MAP3, P5-NT3, LL14, BP121, L-K6 L5W, T-1CEb, CT-K3K7, Lt-MAP2, and CRAMP-18 E2K. These similarities suggest that our generated peptides could be considered novel AMPs (Table S3). Peptide OrP1 is the only one that shows similarity with a natural peptide, Osmin (47.6%), derived from the venom of the solitary bee *Osmia rufa* [37]. The highest similarity percentage was found in VeP1, with 66.7% similarity to the synthetic peptide CT-K3K7, derived from amino acid substitutions in a natural scorpion peptide [38].

The 12e synthesized peptides were evaluated in bioassays to determine their effectiveness against bacteria, *C. albicans*, and cancer cells. Most of the peptides (91.7%) of this study showed MICs against 1 or 2 bacterial species below 16 µM, higher than peptides reported in similar studies that analyzed proteomes or transcriptomes [14, 39]. Bolatchiev et al. [40] employed RNN with LSTM cells to generate five peptides, of which two (of 28 and 32 amino acids) showed antibacterial activity against *K. pneumoniae* with a MIC between 0.5 and 2.7 µM, and only one showed activity against *P. aeruginosa* with a MIC of 0.5 µM, achieving a successful peptide rate of 40%. On the other hand, Tucs et al. [41] implemented a GAN model to generate 7 peptides, of which 4 demonstrated activity against *E. coli* with a MIC below 10 µM, achieving an effectiveness of 57.1% with sequences of 19 to 20 amino acids long. The results obtained in the present work are comparable since 50% of the peptides generated using artificial intelligence models (3 out of 6) showed a MIC lower than 10 µM against at least 1 bacterial species but with size between 12 and 15 amino acids, which shows that the methodology presented is more effective and scalable. Furthermore, the efficiency of the modified peptides increased to 100%, where all 6 peptides exhibited a MIC lower than 10 µM against at least 2 bacterial species, with lengths equal to or shorter than the original peptide sequence.

Peptide VeP1 showed activity against *E. coli* and *S. aureus* with MICs below 10 µM and cytotoxicity to A549 and MCF-7 cells with an IC_50_ below 7 µM. However, it was also the most hemolytic, causing 55.6% hemolysis at 50 µM (Table 3). The amino acid sequence of VeP1 defines a hydrophilic face composed of four K’s interrupted by a W and a hydrophobic face with four L’s and three F’s, characteristics associated with hemolytic peptides [23]. Peptides OrP1M, OrP3M, and OrP4M stand out for being short (14 and 15 amino acids), polycationic (charges of + 5 and + 6), and possessing hydrophobicity between 57 and 60%, desirable properties in the rational design of AMPs [42]. These peptides showed MICs in the range of 1.9 to 15.8 µM against at least 4 microorganisms and cytotoxicity against the MCF-7 cell line with an IC_50_ in the range of < 6.25 to 7.4 µM. The electrostatic interactions of cationic anticancer peptides are related to the alteration of the tumor cells’ membrane charge due to hypoxia and high levels of reactive oxygen species, which expose anionic phosphatidylserine on the outer layer. In addition, the dysregulation of glycosylation and the overexpression of heparan sulfate proteoglycans contribute to the negative charge of the tumor cell membrane [43].

Although some of the peptides evaluated in this study exhibited relatively high MIC values, exceeding 32 µM, it is important to highlight that 11 of the peptides generated demonstrated antimicrobial activity against at least one bacterial strain with MICs below 16 µM. When compared with previous studies, it becomes evident that concentrations within the 10–20-µM range are commonly observed among synthetic AMPs and are considered effective, while also displaying low cytotoxicity in cellular models. For instance, the NRC-16 peptide showed significant inhibition of biofilm formation and antimicrobial activity against *Pseudomonas aeruginosa* and *Staphylococcus aureus* at concentrations ranging from 4 to 16 µM, without evidence of relevant cytotoxicity [44]. Similarly, the TP peptide, derived from Tibetan swine, exhibited activity against both Gram-negative and Gram-positive bacteria with MICs between 2.5 and 20 µM, alongside controlled cellular toxicity [45]. The synthetic peptide 16-PLL10 was also effective at concentrations as low as 1.25 µM, although it showed increased cytotoxicity in human cells at therapeutic doses [46].

These findings suggest that the peptides developed in this study fall within a functional range comparable to other sequences with recognized therapeutic potential. Nevertheless, additional rounds of structural optimization including sequence truncation, cyclization, amino acid substitution, or chemical stabilization using D-amino acids, may further enhance antimicrobial potency and reduce the effective concentrations required, as has been demonstrated in previous peptide development efforts [47–49].

The helical wheel representation of peptides OrP1M, OrP3M, and OrP4M, determined with HeliQuest (data not shown, [20]), indicates the presence of two defined faces, one with WLLL, VLLL, and ILLL residues, respectively, and a hydrophilic positive face formed by RKKKK residues, in the three peptides, configuration that is typical of peptides with antimicrobial and anticancer activity [50]. Amphipathicity favors insertion into the membrane, increasing cytotoxicity due to their affinity for zwitterionic membranes such as those of tumor cells [42].

Four peptides (OrP1M, OrP3M, OrP4M, and OrP9M) with broad-spectrum antimicrobial activity were identified, showing efficacy against at least two microorganisms, whether bacteria or yeast, with MICs below 10 µM and against cancer cells with an IC_50_ below 8 µM. These peptides caused less than 10% hemolysis in human erythrocytes at 50 µM (Table 2). They were generated with AmPepGen [16] and subsequently modified, suggesting that the combined methodology allows the production of broad-spectrum peptides whose mechanism of action can overcome differences in the membrane composition of *C. albicans*, *S. aureus*, Gram-negative bacteria, and cancer cells.

The antimicrobial activity of the peptides OrP1M, OrP3M, OrP4M, OrP9M, and VeP1 was analyzed using scanning electron microscopy. It was observed that the treated cells were smaller than untreated, showed loss of characteristic morphology, and exhibited evident cellular damage, including loss of turgor, blister formation, wrinkles and grooves in the membrane, cell destruction, and leakage of cytoplasmic material. After treatment with the selected peptides, damage to the microorganism’s membranes was widespread, and released cytoplasmic material was observed. Peptide OrP3M, in the three bacteria and yeast tested, caused the loss of cell shape and induced membrane lysis, releasing cytoplasmic material. On the other hand, peptide OrP9M induced the formation of wrinkles and blisters in the membranes of *C. albicans* and *P. aeruginosa* cells (Fig. 2). 

## Conclusion

In conclusion, the combined computational strategy employed in this study proves to be effective in the generation and design of novel potent antimicrobial peptides with defined physicochemical characteristics, since 11 out of the 12 peptides evaluated were active against at least 1 bacterial species or yeast at concentrations ranging from 1.8 to 15.8 µM, and seven peptides showed activity against at least 3 bacterial species and *C. albicans* at concentrations from 7.8 to 15.8 µM. In addition, 6 peptides were effective against at least 1 cancer cell line at concentrations ranging from < 6.25 to 9.1 µM. This combined strategy has improved our ability to predict and to design a set of broad-spectrum peptides and ensure that a large majority exhibit antimicrobial activity at low concentrations, with biosafe characteristics for normal eukaryotic cells.

## Supplementary Information

Below is the link to the electronic supplementary material.

Supplementary file 1 (DOCX 1729 KB)
