# Supplementary material for: Antimicrobial Peptides Design Using Deep Learning and Rational Modifications: Activity in Bacteria, Candida albicans, and Cancer Cells
Source: Curr Microbiol. 2025 Jul 11;82(9):379. doi: 10.1007/s00284-025-04346-3 (PMC12254070; doi:10.1007/s00284-025-04346-3)
Supplement: Supplementary file 1 — (DOCX 1729 KB) [file 284_2025_4346_MOESM1_ESM.docx]

**Supplementary information**

**Table S1**. Prediction of carcinogenic activity and membrane binding probability with machine learning algorithms, of generated and modified synthetic peptides.

| **Peptide name** | **Prediction of anticancer activity^a^** | **Prediction of membrane binding probability^b^** | | |
| --- | --- | --- | --- | --- |
|  | **ENNAACT** | **AMPHIPASEEK** | | |
|  | **DNN** | **Min** | **Max** | **Classification** |
| OrP1 | 0.197 | -0.203 | 0.273 | No binding |
| OrP1M | 1.000 | 0.105 | 0.528 | High binding |
| OrP3 | 1.000 | -0.044 | 0.395 | No binding |
| OrP3M | 1.000 | 0.114 | 0.619 | High binding |
| OrP4 | 0.999 | -0.082 | 0.357 | No binding |
| OrP4M | 1.000 | 0.412 | 0.792 | High binding |
| OrP9 | 0.998 | -0.094 | 0.311 | No binding |
| OrP9M | 1.000 | 0.180 | 0.611 | High binding |
| VeP1 | 1.000 | 0.411 | 0.965 | High binding |
| VeP1M | 1.000 | 0.287 | 0.754 | High binding |
| VeP2 | 0.989 | 0.024 | 0.448 | Binding |
| VeP2M | 1.000 | 0.239 | 0.702 | High binding |
| Random^+^ | N.A. | -0.802 | -0.524 | No binding |
| SlP20* | N.A. | 0.088 | 0.324 | High binding |

Abbreviations: DNN, Deep neural network; Min, Minimum; Max, Maximum. N.A. Not available

^a^ Prediction of estimated anticancer activity with the algorithms available in ENNAACT [24].

^b^ Estimated probability of membrane binding predicted with AmphipaSeeK [25].

* Positive control SIP20, obtained from Monsalve et al. [15].


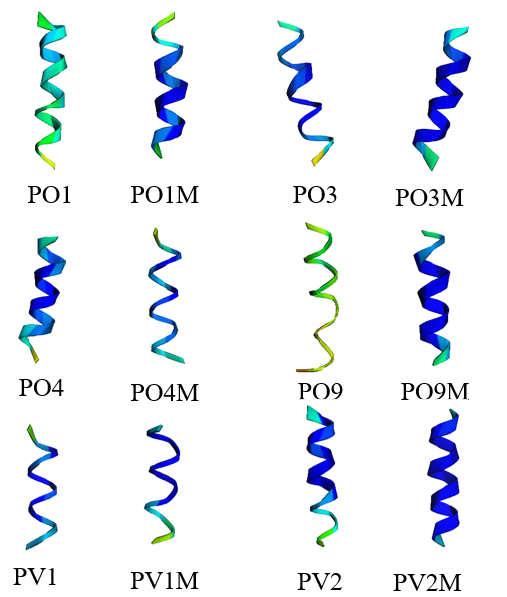

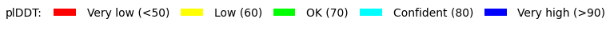


**Figure S1.** 3D structural model of the original and modified peptides, generated with AlphaFold-2 using the ColabFold tool created by Mirdita et al. [36].

With the aim of optimizing peptide interaction with bacterial membranes, a rational design strategy was employed using HeliQuest [20] to improve the spatial arrangement of residues in the helical wheel, promoting a clear segregation between hydrophobic and polar faces. This amphipathic organization is critical for effective insertion into the lipid bilayer. In parallel, Type-Peptide 1 [21] was used to monitor physicochemical properties in real time, ensuring an appropriate balance between charge, hydrophobicity, and secondary structure. As a result, rationally improved peptides were obtained with enhanced functional profiles.

| 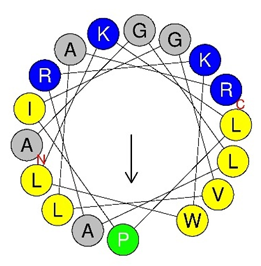 | 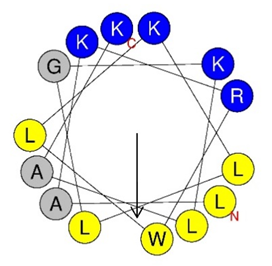 | 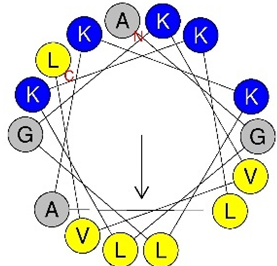 | 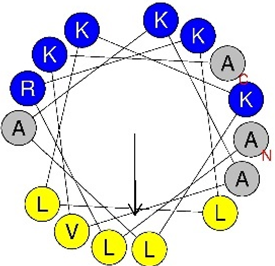 |
| --- | --- | --- | --- |
| PO1 | PO1M | PO3 | PO3M |
| 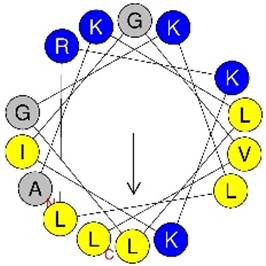 | 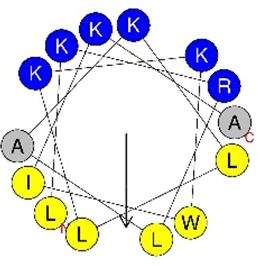 | 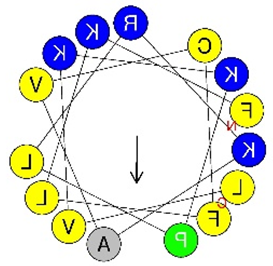 | 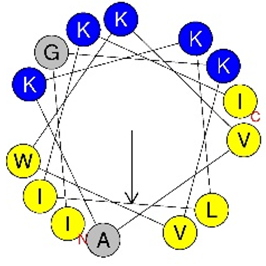 |
| PO4 | PO4M | PO9 | PO9M |
| 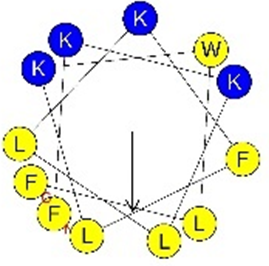 | 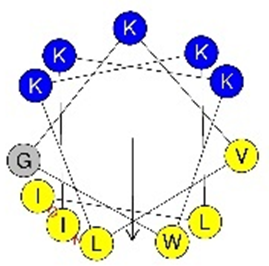 | 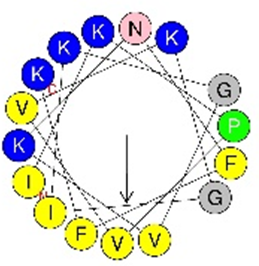 | 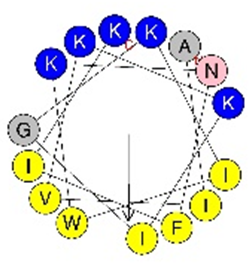 |
| PV1 | PV1M | PV2 | PV2M |

**Figure S2.** Helical wheel projections of original and modified peptides generated using HeliQuest [20], for the rational selection of amino acid residues to be modified.

**Table S2**. Characterization of the synthesized peptides: Molecular weight by Mass Spectrometry and purity by HPLC.

| **Peptide name** | **MW by MS** | **Purity by HPLC** |
| --- | --- | --- |
|  | **Da** | **%** |
| OrP1 | 1861.05 | 77.62 |
| OrP1M | 1637.20 | 98.73 |
| OrP3 | 1564.50 | 86.87 |
| OrP3M | 1649.60 | 83.15 |
| OrP4 | 1649.10 | 97.86 |
| OrP4M | 1707.60 | 88.17 |
| OrP9 | 1789.20 | 93.16 |
| OrP9M | 1623.20 | 97.22 |
| VeP1 | 1609.60 | 88.38 |
| VeP1M | 1452.60 | 94.81 |
| VeP2 | 1800.80 | 80.43 |
| VeP2M | 1785.20 | 97.82 |

MW by MS: Molecular weight by Mass Spectrometry, expressed in daltons (Da or g/mol).

HPLC: High Performance Liquid Chromatography.

**Table S3.** Percentage of similarity of the generated and modified peptides with peptides from the APD3 database, their source and reported activity. The underlined amino acids indicate the modifications made to the original generated sequence.

| **Peptide name** | **Sequence** | **ID APD3: % similarity ^a^** | **Source of the APD3 peptide** | **Reported activity of the APD3 peptide** |
| --- | --- | --- | --- | --- |
| OrP1 | AGLARKWLKLPIGVLAR | AP02882: 47.6 % | *Osmia rufa* venom | Anti-Gram+ & Gram- Antifungal |
| OrP1M | __LAKRWLKLL_GKLAK | AP00506: 62.5 % | Synthetic | Anticancer |
| OrP3 | AGLKKLAKKLGKVVL | AP03635: 56.3 % | Synthetic | Anti-Gram+ & Gram- |
| OrP3M | ALRKLLKKLAKAVKA | AP00506: 62.5 % | Synthetic | Anticancer |
| OrP4 | AKLLGKLLRKKIGVL | AP03675: 56.3 % | Synthetic | Anti-Gram+ & Gram- |
| OrP4M | LKRLAKLLKKWIKA_ | AP03825: 60.0 % | Synthetic | Anti-Gram+ & Gram- Antifungal |
| OrP9 | FKLFCVAKRLPKKVL | AP04180: 53.3 % | Synthetic | Anti-Gram- |
| OrP9M | IGKVWKVAKKLIKI__ | AP04585: 53.3 % | Synthetic | Anti-Gram+ & Gram- |
| VeP1 | FKKLLKFLKWLF | AP00143: 66.7 % | Synthetic | Anti-Gram+ & Gram- |
| VeP1M | IKKWGKVLKKLI | AP03510: 64.3% | Synthetic | Anti-Gram+ & Gram-, Antibiofilm, Anticancer |
| VeP2 | IKPVVKGIKGVKNFFK | AP03631: 47.4 % | Synthetic | Anti-Gram+ & Gram-, Antifungal, Candidacidal, Anticancer |
| VeP2M | _AIVKKIGKIWKNFIK | AP03631: 52.6 % | Synthetic | Anti-Gram+ & Gram-, Antifungal, Candidacidal, Anticancer |

^a^ Percentage of similarity calculated with Antimicrobial Peptide Designer in APD3: Antimicrobial Peptides Database [11].

A total of 36 peptides were selected, including computationally designed sequences and antimicrobial peptides previously reported in the APD3 database [11]. Sequences were organized in FASTA format and aligned using the MUSCLE algorithm implemented in MEGA X version 10.2.6 [51].

The phylogenetic tree was constructed using the Maximum Likelihood method based on the Jones-Taylor-Thornton (JTT) substitution model. Initial trees for the heuristic search were automatically generated by applying the Neighbor-Joining and BioNJ algorithms to a matrix of pairwise distances estimated under the JTT model, and subsequently selecting the topology with the highest log likelihood value.

The analysis was conducted using the complete deletion option to eliminate all positions containing gaps or missing data. The final dataset included 7 aligned positions. The robustness of the resulting tree was evaluated through a Bootstrap analysis with 1000 replications. All evolutionary analyses were performed in MEGA X.


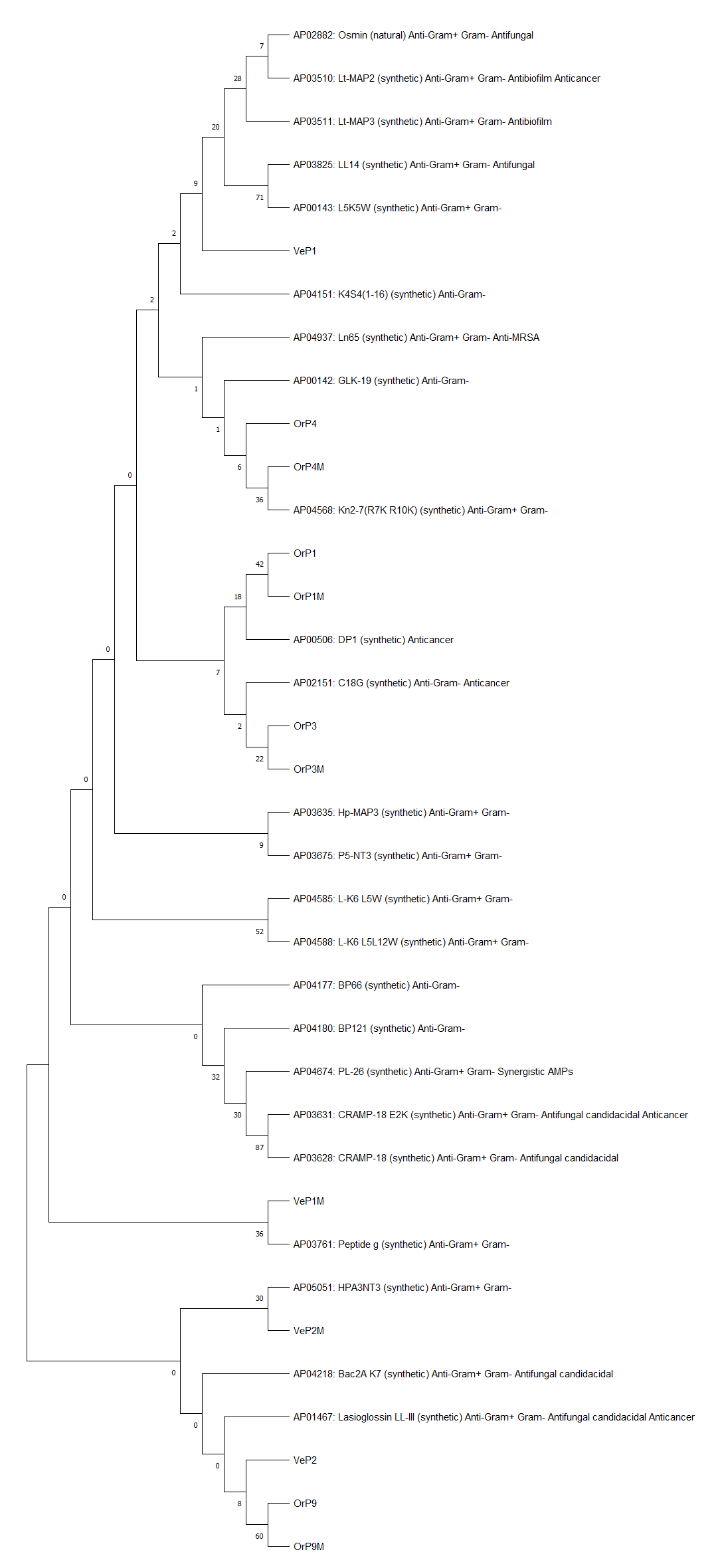


**Figure S3.** Phylogenetic tree of designed and reference antimicrobial peptides based on amino acid sequence similarity**.**

**Table S4.** Half inhibitory concentration (IC₅₀, µM) values of original and modified peptides against pathogenic microorganisms.

| **Peptide name** | **IC_50_ (µM)** | | | | |
| --- | --- | --- | --- | --- | --- |
|  | **C.a.** | **E.c.** | **K.q.** | **S.a.** | **P.a.** |
| OrP1 | 14.8 | 5.8 | 22.4 | 24.2 | 14.8 |
| OrP1M | 10.1 | 1.5 | 4.5 | 2.3 | 10.1 |
| OrP3 | 9.6 | 1.6 | 6.0 | 10.1 | 9.6 |
| OrP3M | 4.6 | 2.4 | 8.6 | 5.3 | 4.6 |
| OrP4 | 23.3 | 14.8 | 29.0 | - | 23.3 |
| OrP4M | 9.4 | 2.2 | 3.3 | 3.4 | 9.4 |
| OrP9 | 44.1 | 4.9 | 93.0 | - | 44.1 |
| OrP9M | 4.9 | 3.2 | 3.8 | 4.5 | 4.9 |
| VeP1 | 31.7 | 5.8 | 5.9 | 2.1 | 31.7 |
| VeP1M | 10.0 | 3.7 | 6.8 | 8.3 | 10.0 |
| VeP2 | 23.7 | 22.8 | 30.7 | 53.2 | 21.9 |
| VeP2M | 5.9 | 1.6 | 9.2 | 7.9 | 5.9 |

IC_50_, Half inhibitory concentration.

Abbreviations of the microorganism species used: C.a., *Candida albicans*; E.c., *Escherichia coli*; K.q., *Klebsiella quasipneumoniae*; S.a., *Staphylococcus aureus*; P.a., *Pseudomonas aeruginosa*.


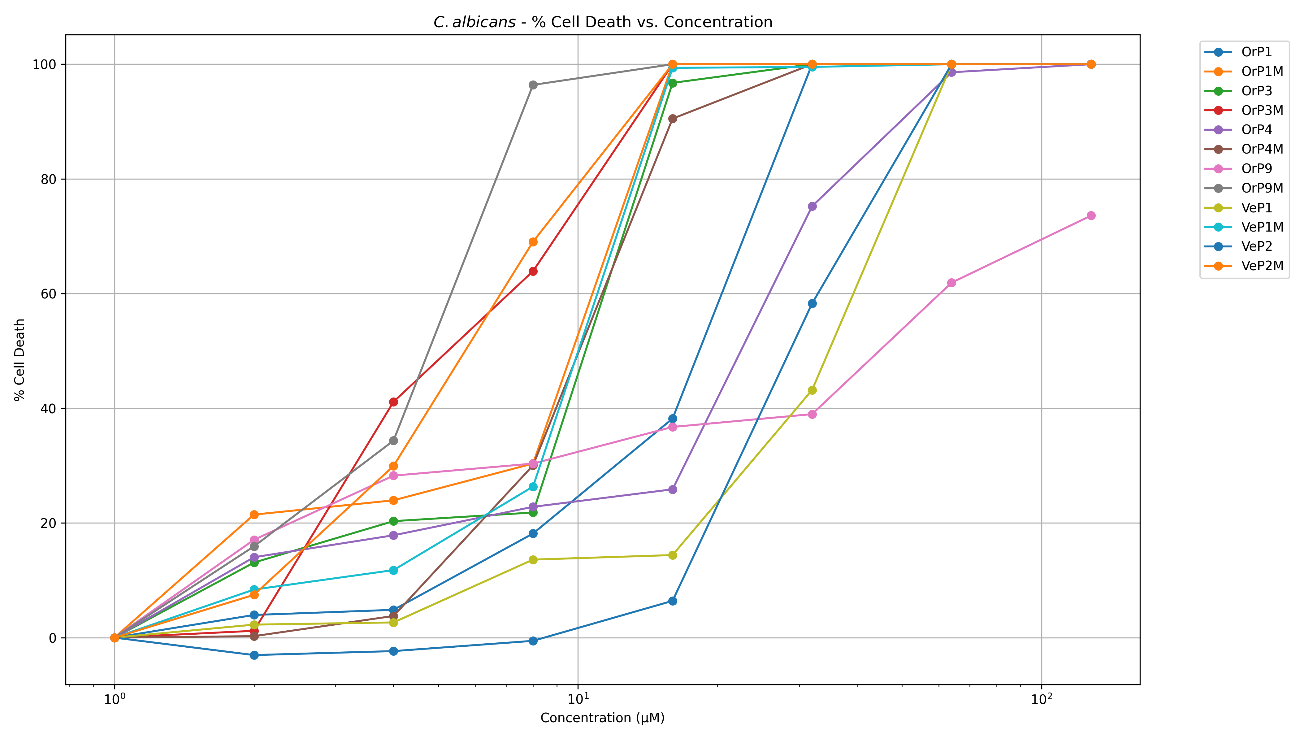


**Figure S4.** Dose-response curves of cell death (percentage) induced by original and modified peptides against *Candida albicans.*


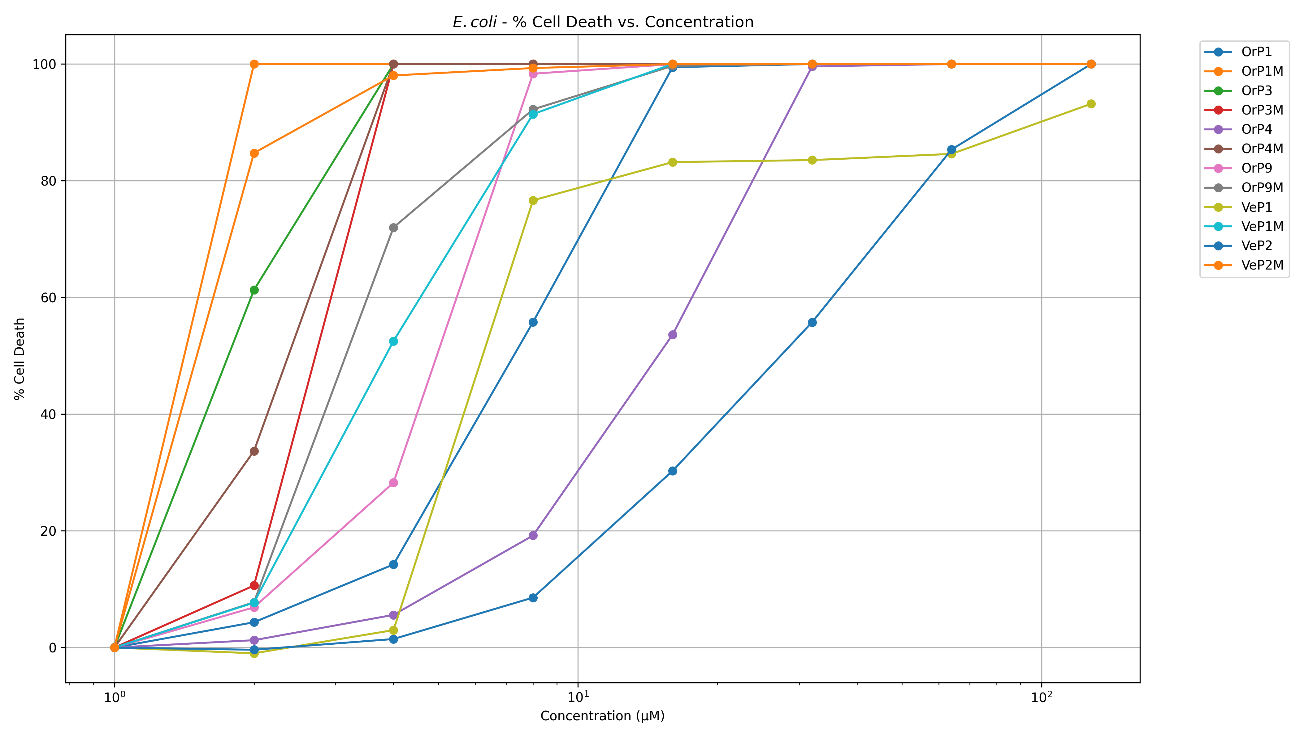


**Figure S5.** Dose-response curves of cell death (percentage) induced by original and modified peptides against *Escherichia coli.*


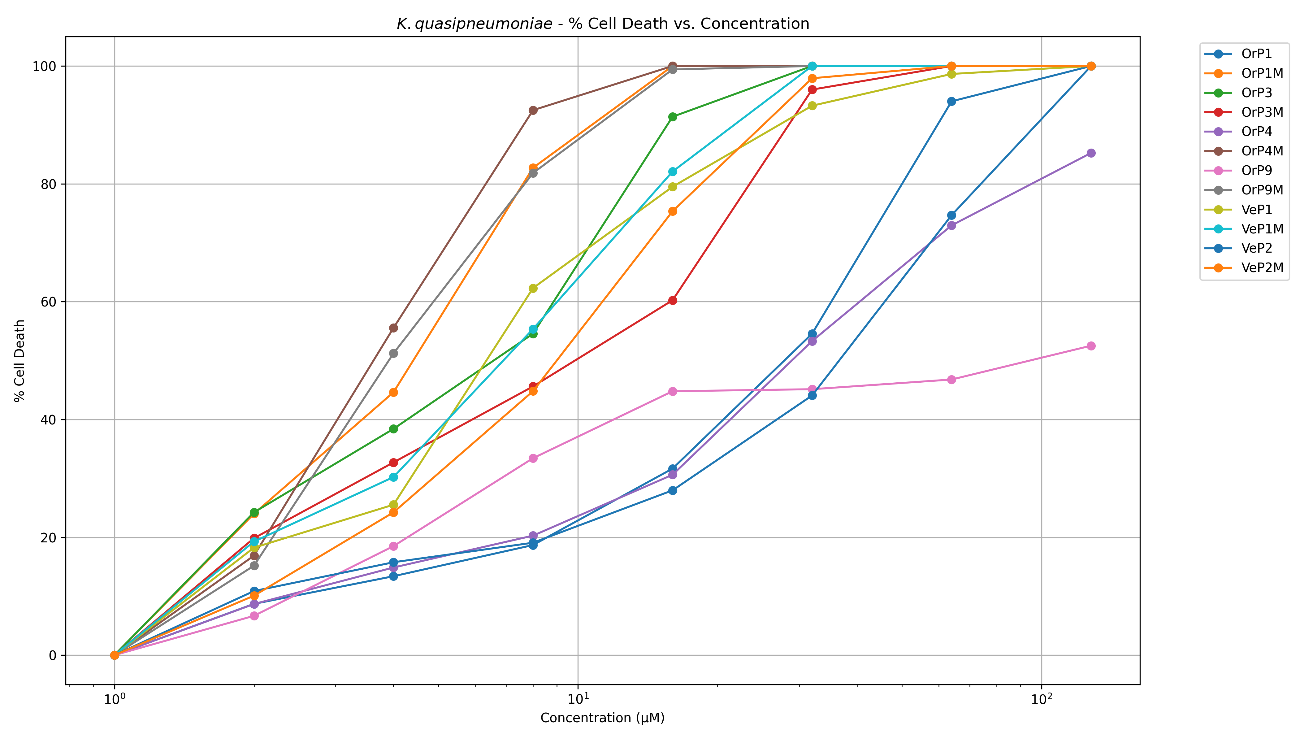


**Figure S6.** Dose-response curves of cell death (percentage) induced by original and modified peptides against *Klebsiella quasipneumoniae.*

**
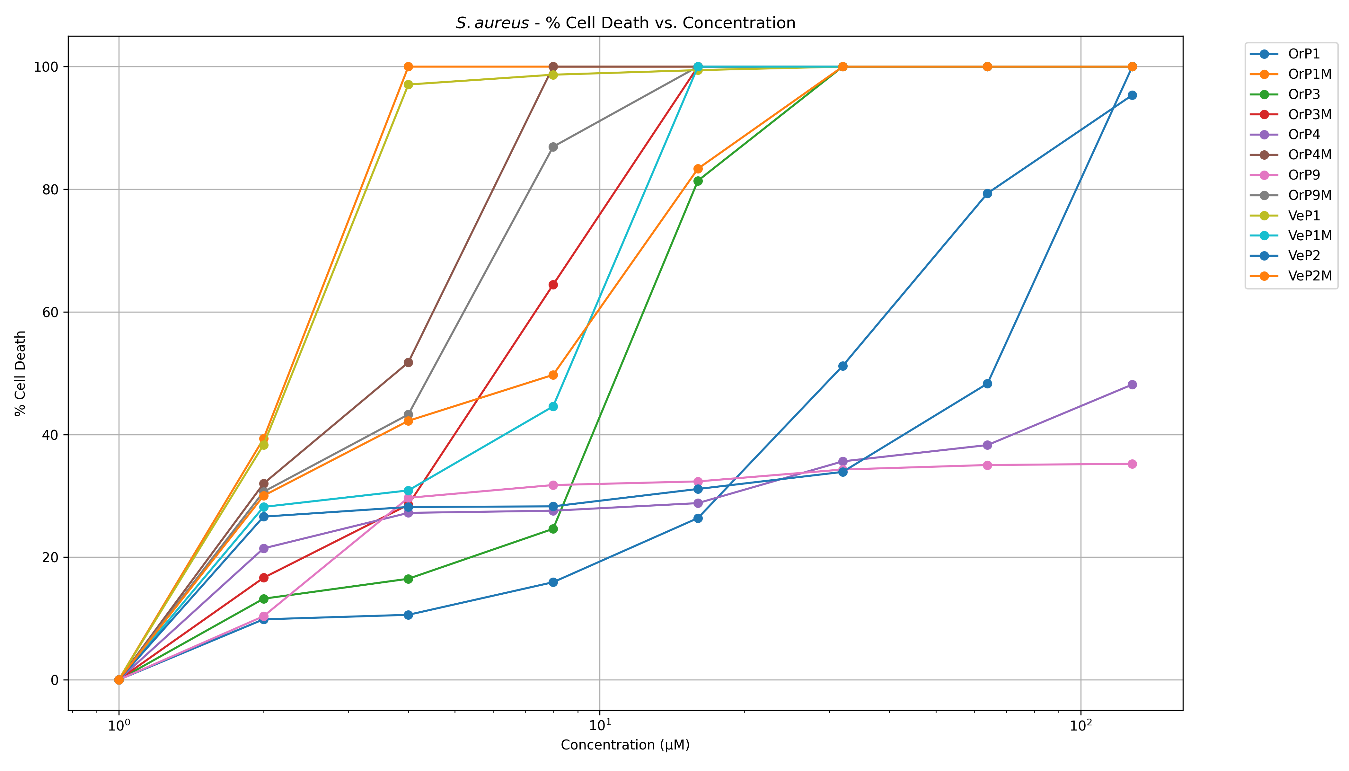
**

**Figure S7.** Dose-response curves of cell death (percentage) induced by original and modified peptides against *Staphylococcus aureus.*

*
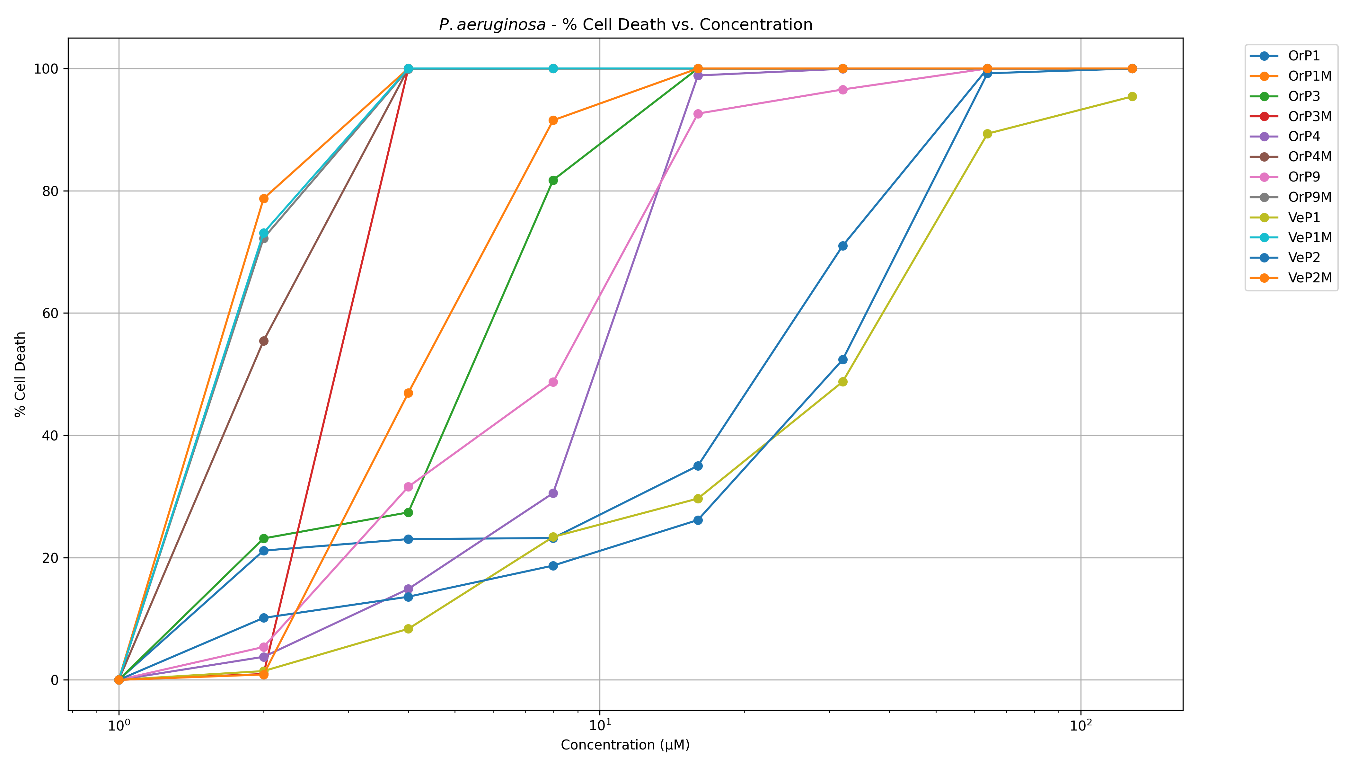
*

**Figure S8.** Dose-response curves of cell death (percentage) induced by original and modified peptides against *Pseudomonas aeruginosa.*

To evaluate the statistical significance of differences in antimicrobial activity between the original peptides and their modified variants, a one-way analysis of variance (ANOVA) was applied to the experimentally obtained minimum inhibitory concentration (MIC) values. Each peptide was tested in four independent replicates against the same target microorganism. Data were grouped according to peptide type (original vs. modified) and compared using ANOVA to determine whether statistically significant differences existed between groups. A p value < 0.05 was considered indicative of statistical significance.

**Table S5.** Statistical comparison of antimicrobial activity between original and modified peptides against five pathogenic microorganisms.

| **Peptide comparison** | **P - Value** | | | | | **General Conclusion** |
| --- | --- | --- | --- | --- | --- | --- |
|  | **C.a.** | **E.c.** | **K.q.** | **S.a.** | **P.a.** |  |
| OrP1-OrP1M | 0.024 | 0.0009 | 2.7×10⁻⁷ | 2.4×10⁻¹² | 2.15×10⁻¹¹ | Activity significantly improved after modification |
| OrP3- OrP3M | **0.356** | **0.356** | **0.0667** | 0.0073 | 0.0025 | Partial improvement against *P. aeruginosa* and *S. aureus* |
| OrP4-OrP4M | 0.0000 | 0.0009 | 5.32×10⁻¹⁵ | 5.32×10⁻¹⁵ | 2.7×10⁻⁷ | Activity significantly improved in all cases |
| OrP9-OrP9M | 0.0031 | **0.134** | 1.86×10⁻⁹ | 1.86×10⁻⁹ | 3.31×10⁻⁸ | Improved activity against all except *E. coli* |
| VeP1-VeP1M | 0.0000 | **0.228** | **0.537** | 3.36×10⁻⁵ | 1×10⁻¹⁰ | Partial improvement; activity decreased against *S. aureus* after modification |
| VeP2-VeP2M | 0.0022 | 2.7×10⁻¹³ | 2.7×10⁻⁷ | 2.7×10⁻⁷ | 1.65×10⁻⁵ | Activity significantly improved in all cases |

Abbreviations of the microorganism species used: C.a., *Candida albicans*; E.c., *Escherichia coli*; K.q., *Klebsiella quasipneumoniae*; S.a., *Staphylococcus aureus*; P.a., *Pseudomonas aeruginosa*.

Statistically significant differences are shown in bold **(p < 0.05).**
